# Ultrasound-Assisted Extraction Coupled with Ion Chromatography for Benzoate Determination in Northern Thai Green Chili Dip: Box–Behnken Optimization, Greenness Assessment, and Application to Commercial Samples

**DOI:** 10.3390/molecules31132259

**Published:** 2026-06-26

**Authors:** Waraporn Chanakul, Nissaya Chuathong, Kanyarak Prasertboonyai

**Affiliations:** Faculty of Science, Energy and Environment, King Mongkut’s University of Technology North Bangkok (Rayong Campus), Rayong 21120, Thailand; waraporn.c@sciee.kmutnb.ac.th (W.C.); nissaya.c@sciee.kmutnb.ac.th (N.C.)

**Keywords:** benzoate, food safety, ion chromatography, response surface methodology, ultrasound-assisted extraction

## Abstract

In this study, an ultrasound-assisted extraction (UAE) method combined with ion chromatography (IC) was developed, optimized and validated for the determination of benzoate in Northern Thai green chili dip. Four extraction variables were optimized using response surface methodology (RSM) based on a Box–Behnken design (BBD). The optimal conditions were a sample mass of 5.0 g, water volume of 20.0 mL, extraction temperature of 70 °C, and extraction time of 20 min. The validated method showed good linearity in the range of 0.5 to 100 mg L^−1^, limits of detection (LOD) and quantification (LOQ) of 0.305 and 1.070 mg L^−1^, respectively, precision in the range of 0.31 to 6.37% (%RSD, n = 11), and spiked recovery in the range of 85.50 to 107.80%, all of which were within the acceptable criteria. The AGREE score of the developed method was 0.45, which was higher than that of the conventional LLE-HPLC-UV method (0.27) due to the use of only deionized water as the extracting solvent and no generation of organic waste. The developed method was sensitive, validated, and environmentally friendly, and was successfully applied to the determination of benzoate in traditional condiment products. The proposed method may serve as an alternative approach for routine food analysis and quality control.

## 1. Introduction

The world food market is growing rapidly, leading to a constant need to search for methods of preservation to increase the food storage time without compromising its safety and quality. The use of preservatives is becoming increasingly important due to the expansion of modern food markets to regional and global levels. Chemical preservatives are one of the most popular means to prolong the shelf life of food by controlling the microbial growth during transportation and storage for a long time. Chemical preservatives are one of the most commonly used additives for ensuring food quality and safety [1]. However, the public concern about the safety of food additives and the enactment of food regulations have made the determination of food preservatives necessary [2].

Benzoic acid (E210) and its salts (sodium benzoate (E211), potassium benzoate (E212), and calcium benzoate (E213) are the most commonly used antimicrobial preservatives in acidic foods such as fruit juices, soft drinks, sauces, pickled food, and condiments [3]. Their use is regulated and permitted only within strictly defined concentration limits established by food safety authorities [4,5]. The mechanism of antimicrobial action of BA involves the penetration of undissociated BA molecules through the microbial cell membrane. Inside the cell, it interferes with microbial metabolism by disrupting of intracellular enzymatic activity and the electron transport chain. This action is pH-dependent because only the undissociated form of BA can penetrate the cell membrane. Therefore, it is more effective at acidic pH (pH ≤ 4.5), where it predominantly exists in its undissociated form [6,7]. The Thai Food and Drug Administration (Thai FDA) has established a maximum permissible limit of 500 mg kg^−1^ for benzoates in condiments and related products. Therefore, reliable analytical methods are required to ensure regulatory compliance and consumer safety in traditional condiments such as Northern Thai green chili dip [8]. Nevertheless, concerns have been raised regarding the possible formation of benzene in benzoate-containing products under specific conditions and the potential biological effects associated with benzoate exposure reported in experimental studies [9,10]. Therefore, the development of a sensitive, accurate, and reliable analytical method for the determination of benzoate in food products is essential for regulatory compliance and food safety monitoring.

Northern Thai Green Chili dip or Nam Prik Noom is a traditional condiment in the Southeast Asian region, especially in Thailand. Nam Prik Noom is composed of dried or fermented ingredients with a high moisture content and an acidic pH, all of which can enhance the risk of microbial spoilage. Consequently, benzoate is frequently added to commercial chili dips to prolong their shelf life and ensure their microbiological safety [11]. A recent food survey on the use of preservatives in Thai condiment sauces has reported that sodium benzoate (INS 211) is a widely used preservative, as indicated by the labeling declaration and its presence in various sauce samples [12]. Since the Thai FDA has set the permissible limits of benzoate as specified by Ministerial Notification No. 418 B.E. 2563 (2020), and some sauces from Asian countries showed preservative non-compliance [8], it is important to develop reliable analytical methods for detecting benzoate in chili dip to meet the regulatory requirements and protect the consumer’s health.

In order to analyze the benzoate in food, sample preparation is one of the key factors for achieving accurate results in food analysis. The target analytes must be effectively extracted from the sample containing complex matrices being introducing to the instrumental analysis. Solvent extraction with organic solvents, alkaline digestion, and liquid–liquid extraction are the most common methods employed for extracting preservatives from food samples [13,14]. However, these techniques are laborious, consume a high volume of organic solvents, and suffer from incomplete extraction or excessive matrix effects, especially when dealing with semi-solid samples, for example, chili dip. Ultrasound-assisted extraction (UAE) has been shown to be an attractive alternative because acoustic cavitation (formation and collapse of bubbles at the solid–liquid interface) can generate localized temperature and pressure gradients that facilitate solvent penetration into the sample, disintegration of the sample, and speed up the transfer of analytes from the sample to the extraction solvent [15]. UAE can shorten the time of analysis and reduce the solvent consumption and energy compared with the conventional methods [16,17]. Furthermore, the use of deionized water as the sole extraction solvent in the present study eliminates organic solvent waste entirely, reinforcing the green credentials of the proposed approach.

Even though, in UAE, all of the aforementioned advantages can be obtained, there are several extraction parameters (i.e., sample weight, solvent volume, extraction temperature and extraction time), which need to be optimized because they are interdependent, such as the effect of temperature that increases the solubility of analyte and the mass transfer rate, but can result in thermal degradation of a heat-sensitive sample, and the effect of solvent volume that increases the extraction capacity, but decreases the analyte concentration in the extract [15]. In the traditional optimization of UAE conditions, one factor is varied at a time, while other factors are kept at a fixed level, which is known as the one-factor-at-a-time (OFAT) methodology. This conventional optimization technique has several drawbacks, such as being time-consuming, and the failure to take into account the interactions among different variables and the possibility of converging to a local maximum rather than a global maximum [17]. The number of runs in the OFAT procedure increases with the number of variables to be studied, which is highly impractical when four or more parameters must be simultaneously considered. This multi-factor optimization can be circumvented by using response surface methodology (RSM), which involves the application of a set of mathematical and statistical techniques to model the relationship between one or more response variables and a set of quantitative or qualitative independent variables [18]. In an RSM design, all the linear, quadratic, and two-factor interaction effects can be assessed simultaneously with a relatively small number of experiments. There are a few RSM designs that can be used for four-factor optimization; however, the Box–Behnken design (BBD) is the most suitable one for this purpose. BBD excludes the extreme factor-level combinations, and only the middle points of the edges of the experimental region are used for the experiment. It also provides a sufficient number of experiments for the estimation of the second-order polynomial model and requires fewer runs in comparison to the full factorial and central composite designs. It has been used in various food analysis studies for optimizing the UAE parameters, and its use in this study will facilitate systematic optimization of the extraction parameters for the extraction of benzoate from chili dip using a minimum number of experiments [19].

After the optimal extraction conditions are obtained, the selection of the analytical detection technique is also crucial for the overall method. Ion chromatography (IC) with conductivity detection is an effective technique for determining benzoate because benzoate is present in the anionic form in the slightly alkaline pH of the carbonate/bicarbonate mobile phase used in anion-exchange separation [20]. It allows for the highly selective separation of the analyte from other neutral and cationic interferences present in the sample matrix. The use of an aqueous IC mobile phase and the aqueous UAE extract (using deionized water as the extraction solvent) offers direct compatibility between the two techniques without the need for solvent exchange, evaporation, or further clean-up before the injection. It can shorten the overall analysis time and decrease the risk of analyte loss. It also contributes to the greenness of the analytical procedure. The previously published IC-based methods for the determination of benzoate in food samples have shown high sensitivity and selectivity for various kinds of foods, including beverages, sauces, and dairy products [21]. However, there is no report on the use of RSM-optimized UAE with IC for the determination of benzoate in semi-solid, paste-like chili-based food samples.

Therefore, this study aimed to: (i) develop and optimize a UAE-based sample preparation method for the determination of benzoate in Northern Thai green chili dip using RSM with a Box–Behnken design, with the four independent variables of sample mass, solvent volume, extraction temperature, and extraction time being evaluated; (ii) validate the analytical performance of the optimized UAE-IC method in terms of linearity, sensitivity, precision, and accuracy; (iii) use the validated method to determine the concentrations of benzoate in commercially available chili dip samples and compare the results with the maximum allowable levels set by national and international authorities; and (iv) use the Analytical GREEnness (AGREE) metric [22], which scores analytical methods from 0 to 1 based on twelve green analytical chemistry criteria, to evaluate the greenness of the proposed method and compare it with the conventional organic-solvent-based extraction methods.

## 2. Results and Discussion

### 2.1. Fitting and Statistical Analysis

The relationship between benzoate concentration and the four UAE extraction parameters was modeled using a second-order polynomial equation derived from the Box–Behnken design. Fitting the experimental data to the quadratic model yielded the following regression equation expressed in terms of coded variables:

$$\begin{array}{l} Y=-4345.240625-\;19.039583X_{1}+\;44.735417X_{2}+\;115.587917X_{3}+\;5.104722X_{4}\\ \;\;\;\;\;\;\;\;-\;5.194792X_{1}^{2}-\;0.641042X_{2}^{2}-\;0.776542X_{3}^{2}-\;0.147907X_{4}^{2}+\;1.658750X_{1}X_{2}\\ \;\;\;\;\;\;\;\;\;+\;1.226250X_{1}X_{3}-\;0.380833X_{1}X_{4}\;-\;0.400250X_{2}X_{3}+\;0.139500X_{2}X_{4}-\;0.000833X_{3}X_{4} \end{array}$$

where X_1_, X_2_, X_3_, and X_4_ denote sample mass (g), water volume (mL), extraction temperature (°C), and extraction time (min), respectively, while Y represents the benzoate concentration expressed on a dry-weight basis (C_dw_, mg kg^−1^).

The adequacy of the models was tested through ANOVA (Table 1). The model F-value of 15.12 with a *p*-value less than 0.001 indicated that the second-order polynomial model was significant and could be used to navigate the design space. The R^2^ value (0.9464) was close to unity, indicating a high degree of correlation between the observed and predicted values, with only 5.36% of the total variation remaining unexplained by the model. The value of the adjusted determination coefficient (adjusted R^2^ = 0.8838) indicated a satisfactory fit of the model [22]. Although the difference between R^2^ (0.9464) and adjusted R^2^ (0.8838) was relatively large, this was attributed to the presence of several statistically non-significant interaction terms (X_1_X_3_, X_1_X_4_, X_2_X_4_, and X_3_X_4_; *p* > 0.05), which contributed little to the predictive performance of the model. To assess the necessity of these terms, model reduction was investigated by removing non-significant effects and comparing the resulting reduced models with the full quadratic model. Model selection was based not only on statistical significance but also on overall model adequacy, predictive capability, and preservation of the hierarchical structure of the response surface model. Since the reduced models did not improve the overall model performance, the complete quadratic model was retained for subsequent analysis and optimization to preserve model hierarchy and ensure reliable response surface interpretation. Furthermore, the non-significant lack-of-fit test (F = 1.05, *p* = 0.582) confirmed that the model was adequate for prediction within the studied experimental range. Overall, the second-order polynomial equation adequately described the relationship between the independent variables and the response and was therefore considered suitable for further analysis.

Among the four linear terms, the effect of sample mass (X_1_) was the most significant (*p* < 0.001) with an F value of 141.55, which was more than 12 times that of the second most significant factor. Extraction time (X_4_; *F* = 11.50, *p* = 0.005) and temperature (X_3_; *F* = 6.83, *p* = 0.023) were also significant, while water volume (X_2_; *F* = 0.06, *p* = 0.810) was found to be insignificant. The significance of the quadratic term X_2_^2^ (*F* = 19.63, *p* = 0.001), X_3_^2^ (*F* = 28.81, *p* = 0.001) and X_4_^2^ (*F* = 5.29, *p* = 0.040) indicated that the quadratic effects of the three variables were significant, which meant there was curvature in the response surfaces of water volume, extraction temperature and time, and the optimal points were within the range of the variables chosen. Only the interaction term of X_2_X_3_ (*F* = 5.74, *p* = 0.034) was significant, which implied that there was an interaction between water volume and extraction temperature. This meant that the effect of water volume on the extraction efficiency depended on the level of extraction temperature.

### 2.2. Effects of Extraction Variables on Benzoate Concentration

**Figure 1:**
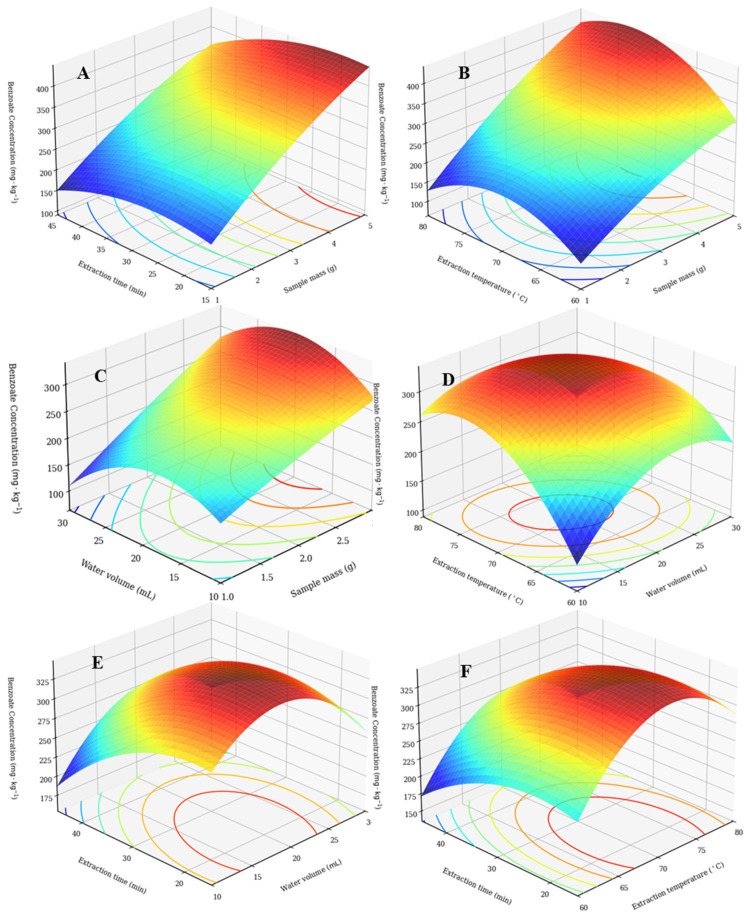
Response surface plots showing the effects of independent variables on benzoate concentration (mg kg^−1^): (**A**) interaction between sample mass and extraction time; (**B**) interaction between sample mass and extraction temperature; (**C**) interaction between sample mass and solvent volume; (**D**) interaction between solvent volume and extraction temperature; (**E**) interaction between solvent volume and extraction time; and (**F**) interaction between extraction temperature and extraction time.

The three-dimensional response surface plots presented in Figure 1 illustrate the individual and interactive effects of the four extraction variables on the response variable, defined as the benzoate concentration in the dried chili dip samples (C_dw_, mg kg^−1^ dry weight). Analysis of variance (ANOVA), together with visual examination of the response surface plots, revealed distinct trends and interaction patterns among the variables. These effects are discussed in detail in the following sections for each extraction parameter.

Consistent with the ANOVA results, sample mass (X_1_) was the most significant factor in all two-variable combinations (Figure 1A–C). Although the response was expressed as benzoate concentration on a dry-weight basis (mg kg^−1^), sample mass strongly influenced the extraction efficiency by altering the solid-to-solvent ratio and mass-transfer conditions during UAE. Increasing the sample mass from 1 to 5 g enhanced benzoate concentration from the matrix, resulting in a corresponding increase in the measured response. The absence of a decline in the response at higher sample masses further suggests that the extraction capacity of the solvent was not exceeded within the investigated experimental range.

Extraction temperature (X_3_) had a significant positive effect on the recovery of benzoate (*p* = 0.023) and indicated that increased extraction temperatures corresponded to increased measured concentrations of benzoate (Figure 1B,D). Several mechanisms can be attributed to the positive effect of temperature, including a decrease in the viscosity of the extraction solvent, increasing its ability to penetrate the pores of the dried chili dip matrix and increasing solvent–matrix interaction; an increase in the aqueous solubility of benzoic acid and sodium benzoate, increasing partitioning to the liquid phase; and an increase in the intensity of acoustic cavitation, which occurs as temperature increases solvent vapor pressure, reducing the critical energy for cavitation initiation and increasing the number of transient cavitation events [23]. The violent collapse of cavitation bubbles creates microstreaming and shearing forces as well as hot-spots (estimated as >5000 K and >500 atm) around the point of collapse that mechanically breaks apart the cell wall and H-bond network structures in the dried chili dip matrix, liberating bound benzoate to the bulk solvent [24]. Cumulatively, these mechanisms result in an increase in the mass transfer rate, consistent with previous observations for UAE at elevated temperatures for the extraction of polar, water-soluble analytes from food. The significant quadratic term for temperature (X_3_^2^; *p* < 0.001) indicates a plateau effect of temperature on benzoate concentration over the studied range, beyond which additional temperature may not result in a significant improvement in extraction efficiency, and may indicate that equilibrium conditions are being approached at elevated temperatures.

Extraction time (X_4_) had a significant positive effect on the measured concentration of benzoate (*p* = 0.005; Figure 1A,E) and indicated that longer extraction times result in higher concentrations of analyte transferred from the solid phase to the liquid phase. The results agree with the intrinsic mechanism of SLE, in which the analyte migration rate from the sample matrix to the solvent is governed by the diffusion across the concentration gradient between the solid phase and the liquid phase [25]. In UAE, the ultrasonic energy renews the concentration gradient at the phase boundary through eliminating the static boundary layer at the solid–liquid interface, providing a driving force for mass transfer far greater than that obtained by traditional extraction and mixing [15]. However, the significant quadratic effect (X_4_^2^; *p* = 0.040) suggests the existence of an optimal extraction time, after which the benzoate concentration in the extract remains nearly constant as the extraction system approaches equilibrium. At this equilibrium state, the rate of analyte desorption from the sample matrix is equal to the rate of re-adsorption, which results in no net mass transfer to the liquid phase [26]. Further prolonging the sonication time can theoretically induce the re-adsorption of analyte on the sample matrix or the thermal decomposition of the analyte in the extract; however, no such phenomena were observed within the range of extraction time examined here.

Although the water volume (X_2_) shows no significant linear effect on the benzoate concentration (*p* = 0.810), the highly significant quadratic term (X_2_^2^; F = 19.63, *p* = 0.001) implies the existence of an optimal level for this parameter within the experimental range. This phenomenon is often observed in those solvent volume-dependent extraction systems: at lower levels of water volume (10 mL), the relatively small water volume in comparison with the sample mass (1–5 g) may not be sufficient to completely dissolve the analyte, especially when larger number of samples was extracted, which resulted in a lower recovery of the analyte [18]. By contrast, at higher levels of water volume (30 mL), the higher water volume diluted the analyte in the extract to result in a lower concentration, although the absolute recovery of the analyte did not change. The two contrary effects resulted in the curvature of the response surface plots of X_2_ (Figure 1C–E), and thus the water volume should be optimized instead of using a relatively large volume of water. The non-significant linear term suggested that at the middle range of water volume, the variation in water volume has relatively small effect on the response variable, as the two contrary effects almost counteracted each other.

The significant interaction effect of water volume and extraction temperature (X_2_X_3_; *F* = 5.74, *p* = 0.034) implies that these two parameters have interactive effects on the extraction of benzoate. The extraction temperature significantly modulated the influence of water volume on benzoate concentration. At elevated temperatures, the increased thermal energy facilitated analyte dissolution and accelerated mass transfer through enhanced diffusion, enabling effective extraction with lower volumes of water. In contrast, lower extraction temperatures limited analyte solubility and diffusion, thereby requiring greater water volumes to generate a sufficient concentration gradient between the sample matrix and the extraction medium. This concentration gradient serves as the driving force for analyte migration, ultimately enhancing extraction performance. The modulation effect of temperature on the optimal water volume is of importance for the robustness of the analytical method. The variation in temperature can influence the optimal water volume for extraction, and thus it is necessary to adjust the water volume in order to keep a constant recovery of analyte when temperature variation occurs in the extraction process. This demonstrates the advantage of RSM approach over the conventional one factor at a time (OFAT) approach for extraction optimization.

### 2.3. Optimization and Validation of UAE Conditions

Optimization of the response surface methodology (RSM) model was performed to maximize the predicted benzoate concentration within the established experimental range. The optimum conditions obtained were 5 g of sample mass (X_1_), 20.8 mL of water volume (X_2_), 73 °C of extraction temperature (X_3_), and 20.42 min of extraction time (X_4_), which resulted in the predicted concentration of 456.62 mg kg^−1^ of benzoate. In order to make it convenient for the experimental conditions, the optimal conditions were changed to 5 g, 20 mL, 70 °C, and 20 min, respectively, and it was not significantly far from the predicted optimal region of the response surface. Three experiments were carried out to validate the modified optimal experimental conditions, and the average concentration of benzoate was found to be 446.72 mg kg^−1^. The prediction error between the predicted and experimental concentration of benzoate was found to be 2.17% and it was less than 5%, which indicates that the quadratic model is reliable and the modified UAE procedure is robust. The predicted and experimental results were in good agreement although the modified temperature (70 °C) and time (20 min) were not exactly the same as the predicted ones (73 °C, 20.42 min), which indicates that the response surface at the optimal region is quite flat and the method is quite robust against the small variation in the experimental conditions, which is one of the advantages of the method for routine analysis where the precise temperature control cannot be easily achieved.

### 2.4. Chromatographic Analysis and Selectivity

**Figure 2:**
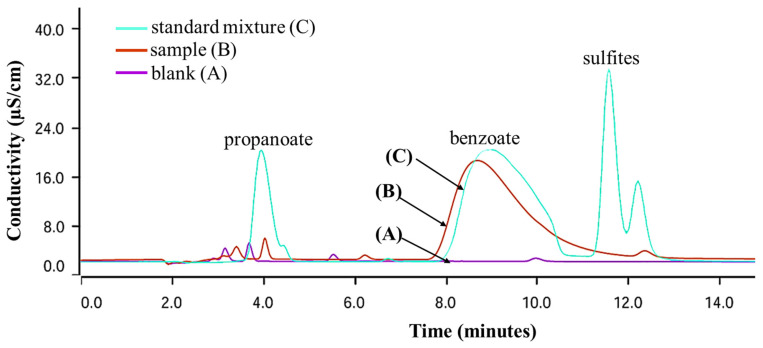
Representative chromatograms of (A) blank, (B) chili dip sample, and (C) standard mixture under optimized ion chromatographic conditions. See Section 3.2.4 for detailed chromatographic conditions.

Figure 2 shows representative ion chromatograms obtained under the optimized IC conditions for three different injections: a reagent blank (A), a chili dip sample extract (B), and a standard mixture (C). The standard mixture chromatogram (C) shows three separated anion peaks for propanoate (t_R_ ≈ 4.1 min), benzoate (t_R_ ≈ 9.13 min), and sulfite (t_R_ ≈ 12.3 min), which eluted in this order from the Metrosep A Supp 5 anion-exchange column with the carbonate/bicarbonate mobile phase system. Although propanoate and sulfite were not target analytes of this study, they are widely used food preservatives that may be present together with benzoate in various condiment products. Therefore, both compounds were also added to the standard mixture to evaluate the selectivity of the Metrosep A Supp 5 column under the experimental conditions. As shown in Figure 2C, baseline resolution was obtained among the three anions, which is a requirement for any analytical method intended to be applied for the determination of benzoate in food samples in which more than one preservative may be present. In addition, in the presence of the three anions, no overlapping between propanoate and benzoate or between benzoate and sulfite was observed. Therefore, the presence of well resolved peaks for the three anions can be used as chromatographic evidence of the method selectivity. In the case of the sample extract (B), a single well-defined symmetrical peak for benzoate was detected with a retention time (t_R_) of 9.13 min, which was very close to the retention time found for the same anion in the standard mixture (Δt_R_ < 0.02 min). In addition to the retention time, the absence of any interfering peak from the sample matrix at or near the retention time of the analyte, as can be observed in Figure 2B, was used to confirm the identity of the peak for benzoate in the sample extract. Figure 2B also shows that the baseline obtained for the sample extract was stable with no significant noise or ghost peaks, and that the peak obtained for benzoate was symmetrical with no tailing. This indicates that the interaction between the analyte and the stationary phase of the analytical column was efficient, and that no significant effect was observed in the peak shape due to the sample matrix. No signal at the retention time of benzoate was observed in the reagent blank chromatogram (Figure 2A), which indicated that no contamination from anions at a concentration level near the LOD of the method was observed due to the membrane filtration procedure, polypropylene centrifuge tubes, or deionized water used to extract the analyte from the sample. Based on the discussion above, the results shown in Figure 2 indicate that the proposed UAE-IC method provided adequate resolution, high selectivity, and a clean baseline for the determination of benzoate in the chili dip sample.

### 2.5. Analytical Performance

The analytical performance of the optimized UAE-IC method is listed in Table 2. The method was linear in the range of 0.5 to 100 mg L^−1^ with R^2^ = 0.9993 and a regression equation of y = 1.251x + 0.076 (y: conductimetric peak area (µS·min), x: concentration of benzoate (mg L^−1^)). The regression coefficient (R^2^) of 0.9993 indicates a linear response between the peak area and concentration of the analyte within two orders of magnitude. This linear range is sufficient to determine the concentration of benzoate in commercial chili dip samples upon proper dilution. The linear range of this method is similar to or wider than those previously reported for IC (1 to 50 mg L^−1^) and HPLC (0.5 to 50 mg L^−1^) methods for determining benzoate in food samples [21,27].

The LOD and LOQ, calculated from S/N of 3 and 10, respectively, were calculated as 0.305 mg L^−1^ and 1.070 mg L^−1^. The LOD and LOQ compare favorably with those reported for IC-based methods applied to similar food matrices. For example, D’Amore et al. reported an LOD of 1.6 mg kg^−1^ and an LOQ of 4.9 mg kg^−1^ for benzoate in food using capillary IC with conductivity detection, while HPLC-UV methods for benzoate in sauces have typically yielded LODs in the range of 1.5–5.0 mg kg^−1^ [21]. The lower LOD and LOQ achieved by the present UAE–IC method can be attributed in part to the efficient and complete extraction of benzoate afforded by the optimized UAE conditions, which maximizes the analyte concentration in the extract, and therefore the signal magnitude, at a given sample benzoate level.

Method precision, expressed as the relative standard deviation (%RSD) of 11 replicate analyses, ranged from 0.31% to 6.37% across the concentration levels evaluated. The lowest %RSD values were observed at the mid-range concentration levels (approximately 5–50 mg L^−1^), consistent with the general principle that instrumental precision is highest in the central portion of the calibration range, away from the LOQ and the upper linearity limit where signal-to-noise is either limiting or detector response may approach nonlinearity. All %RSD values were below the 10% threshold recommended by AOAC guidelines for food analytical methods at analyte levels above the LOQ, and the majority (>80%) were below 5%, a more stringent but commonly applied precision criterion in food safety applications. The intra-day and inter-day %RSD values were comparable, indicating that the method is robust against temporal variation in laboratory conditions including minor fluctuations in column temperature, mobile phase composition, and instrument baseline drift.

Method accuracy was evaluated through recovery experiments in which pre-analyzed chili dip samples were spiked with benzoate at 20 mg kg^−1^ prior to extraction. Recoveries for the five commercial samples ranged from 85.50% to 107.80% (Table 3), all within the AOAC acceptance range of 80–110% for analytes at this concentration level [28]. These results demonstrate satisfactory extraction efficiency and method accuracy across different sample matrices. The absence of a consistent recovery bias, with values both above and below 100%, indicates that the observed variations were primarily due to matrix-specific compositional differences rather than systematic methodological bias.

The developed UAE–IC method proved to be accurate, robust, and environmentally friendly, owing to the use of deionized water as the extraction solvent. The method was successfully applied to the determination of benzoate in Northern Thai green chili dip products. However, as the present study represents a single-laboratory validation based on five commercial samples of a single food matrix and one spike level (20 mg kg^−1^), further validation involving multiple laboratories and additional high-matrix sauce and condiment products is recommended before routine implementation in food safety monitoring programs.

### 2.6. Comparison with Other Available Methods

To place the performance of the proposed method in context, its analytical performance and greenness characteristics were compared with those of recently reported methods for the determination of benzoate (and the co-occurring preservative sorbate) in food matrices (Table 4). The developed UAE-IC method, which uses only deionized water as the extraction solvent, provided an LOD of 0.305 mg L^−1^, an LOQ of 1.070 mg L^−1^, and recoveries ranging from 85.50% to 107.80%. These figures are comparable to, or better than, those reported for capillary ion chromatography with conductivity detection (LOD 4.1 mg kg^−1^; recovery 90.2–105.8%) [21] and solid-phase extraction coupled with ion chromatography for milk analysis (recovery ≈ 90–105%) [20]. Compared with a recently reported green reversed-phase HPLC method combined with ultrasound-assisted extraction (recovery 84.0–100.8%) [29] and a headspace GC–MS method for sauces and pastes [30], the proposed approach offers comparable sensitivity and accuracy while completely eliminating the use of organic solvents. Furthermore, the aqueous extract is directly compatible with the IC mobile phase, avoiding the solvent evaporation and reconstitution steps commonly required in liquid–liquid extraction-based HPLC procedures.

These advantages are evidenced by the higher AGREE greenness score achieved by the proposed method (0.45) compared with the conventional LLE–HPLC–UV approach (0.27). A comprehensive criterion-by-criterion comparison, encompassing the extraction method, solvent consumption, limits of detection (LOD), recovery, and precision, is presented in Table 2.

### 2.7. Greenness Evaluation Using the AGREE Metric

**Figure 3:**
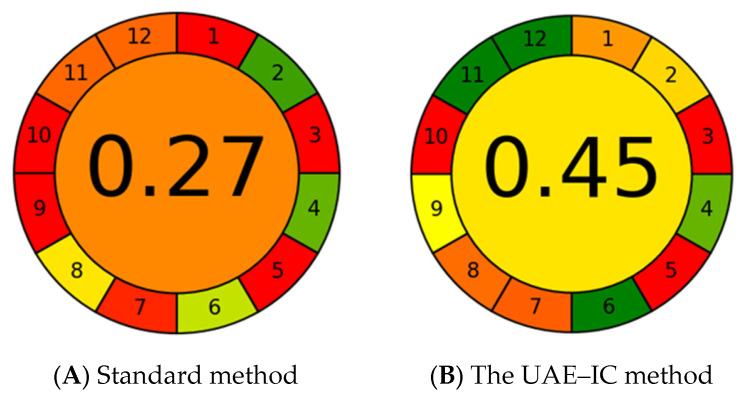
AGREE Metric value for benzoate measurements.

To quantitatively compare the environmental friendliness of the presented UAE-IC method and a conventional method used for benzoate determination in food samples, the Analytical GREEnness (AGREE) tool was used. AGREE compares the greenness of an analytical procedure to twelve principles of green analytical chemistry. The twelve principles include: sample throughput, in-line or direct analysis capability, derivatization requirements, hazardous reagent consumption, waste generation, operator exposure risk, energy consumption, method miniaturization, and the capacity for multi-analyte detection. A score of 0 to 1 is obtained for each principle depending on how green the analytical procedure is with respect to each principle. The overall greenness of the method is calculated as a composite score between 0 and 1, where higher values denote a more environmentally favorable method. For comparison purposes, AGREE was calculated for both the developed UAE-IC method and a conventional liquid–liquid extraction (LLE) procedure followed by HPLC-UV determination (conventional method) [33,34]. Figure 3 displays the calculated clock face pictograms with corresponding AGREE scores for both methods.

The calculated AGREE score for the UAE-IC method was 0.45, while the score for the LLE-HPLC-UV method was 0.27. This corresponds to a 67% relative improvement in the greenness of the developed method compared to the conventional one. The most significant improvements are seen in the use of hazardous reagents (Principle 5) and the generation of waste (Principle 6). The use of deionized water as the sole extraction solvent prevents the consumption of toxic reagents such as acetonitrile, methanol, or dichloromethane commonly employed in LLE procedures. Additionally, the use of a diluted carbonate/bicarbonate buffer as the IC mobile phase compared to organic gradients typically used in RP-HPLC methods decreases the overall amount of hazardous chemical waste produced per analysis. Since the aqueous UAE extract is directly compatible with the IC mobile phase, no solvent evaporation and reconstitution steps are required. This absence of evaporation contributes positively to Principles 3 (direct analysis capability) and 7 (operator exposure) because the evaporation step can expose the analyst to harmful solvent vapors and consumes energy. The absence of chemical derivatization in both extraction and detection steps also maximizes the score for Principle 4 (derivatization). Finally, the use of conductivity as the detection technique, a reagentless detection mode, prevents the use of chromogenic or fluorogenic reagents, further minimizing chemical waste production and decreasing reagent costs. The complete score and justification for each of the twelve AGREE criteria, for both the UAE–IC and the conventional LLE–HPLC–UV methods, are provided in the Supporting Information (Table S1).

Even though the absolute AGREE score obtained for the developed method is still far from the value of 0.75 that has been suggested to indicate a truly green analytical procedure [22,35], this is unsurprising for a method for analysis of semi-solid, non-homogeneous food samples. The AGREE score of the developed method is reduced by the need to analyze relatively large sample sizes (5 g; Principle 2), perform off-line sample pretreatment, which includes drying, weighing, and centrifugation steps (Principle 3), and use a non-automated extraction technique (Principle 8). Furthermore, the energy needed to power the ultrasonic bath during extraction and the IC system during analysis (pump, conductivity detector, and column oven) slightly decreases the score for Principle 9 (energy saving), although the short duration of the extraction (20 min) and the absence of lengthy column conditioning periods minimize the impact of this drawback. Although the complexity of the semi-solid, heterogeneous chili-dip matrix partly necessitates these experimental conditions, they nevertheless represent limitations of the present method that contribute to the moderate AGREE score. Off-line drying, weighing, and centrifugation, together with the energy demand of the ultrasonic bath and IC system and the relatively large sample mass (5 g), negatively affect the greenness assessment. These limitations could potentially be mitigated in future studies through miniaturized or automated sample handling, reduced sample and solvent consumption, and lower-energy extraction approaches, provided that the analytical performance demonstrated in Section 2.5 is maintained. Consideration of these limitations provides a more balanced and comprehensive evaluation of the overall greenness of the analytical procedure.

Overall, the AGREE evaluation highlights that the presented UAE-IC method offers a practical green advancement for the analysis of food preservatives. The 67% improvement in the calculated AGREE score of the developed method compared to the conventional one translates into an appreciable reduction in the consumption of organic solvents, production of hazardous waste, and exposure of the operator to chemicals per individual analysis. The environmental gains are especially significant in routine analysis laboratories where dozens or even hundreds of samples are analyzed on a monthly basis.

### 2.8. Application to Real Chili Dip Samples

The applicability of the developed UAE–IC method was evaluated by analyzing five Northern Thai green chili dip samples. Benzoate was detected in all samples, with concentrations ranging from 263.00 to 501.07 mg kg^−1^ on a dry-weight basis (C_dw_) and from 54.33 to 103.23 mg kg^−1^ on a wet-weight basis (C_ww_) (Table 3). The highest benzoate concentration was observed in Chili dip 2, whereas Chili dip 4 contained the lowest level. The observed variation among samples may reflect differences in formulation, ingredient composition, and manufacturing practices among producers.

For regulatory assessment, wet-weight concentrations (Cww) are the most relevant because food additive limits are established for the final product as consumed. The Codex Alimentarius Commission (CAC) and the Thai Food and Drug Administration (Thai FDA) specify a maximum permissible limit of 500 mg kg^−1^ for benzoates in condiments and related products. All analyzed samples contained benzoate concentrations well below this limit, ranging from 54.33 to 103.23 mg kg^−1^, corresponding to approximately 10.9–20.6% of the maximum permitted level. These findings indicate that all tested products complied with the current regulatory requirements.

The low variability among replicate measurements further supports the reliability of the developed method when applied to complex condiment matrices. Taken together, these results demonstrate the suitability of the UAE–IC method for the routine determination and monitoring of benzoate in Northern Thai green chili dip products.

## 3. Materials and Methods

### 3.1. Chemicals and Reagents

The reagents used in this work were of analytical reagent (AR) grade. Benzoic acid (benzoate reference standard; Sigma-Aldrich, St. Louis, MO, USA), anhydrous sodium carbonate (Na_2_CO_3_; Sigma-Aldrich, St. Louis, MO, USA), and sodium bicarbonate (NaHCO_3_; Sigma-Aldrich, St. Louis, MO, USA) were used in this study. Ultrapure water (resistivity ≥ 18.2 MΩ cm^−1^ at 25 °C), from a Milli-Q water purification system (Millipore, Bedford, MA, USA), was used for the preparation of all solutions, eluents, and sample dilutions. A 100 mg L^−1^ benzoate working stock solution was prepared by accurately transferring 10.00 mL of a 1000 mg L^−1^ certified benzoate standard solution into a 100.00 mL Class A volumetric flask and made up to volume with ultrapure water. It was stored in an amber glass bottle at 4 °C and used within one week of preparation to prevent degradation and ensure stability. Calibration working standards of appropriate concentration levels were freshly prepared by serial dilution of the stock solution with ultrapure water before each run.

### 3.2. Methods

#### 3.2.1. Sample Collection and Preparation

Five samples of different commercial northern Thai green chili dips (Nam Phrik Noom) were obtained from local retail markets in Rayong Province, Thailand. These samples were chosen to provide a representative selection of the commercially available products in the region. The samples were homogenized prior to analysis. A 100 g portion of the homogenized fresh sample was dried in an oven at 60 °C for 12 h until a constant weight was achieved to reduce moisture content and simplify the sample matrix prior to extraction. The initial moisture content of the samples ranged from 78% to 81%. The dried samples were stored in airtight containers at room temperature until further analysis.

**Scheme 1:**
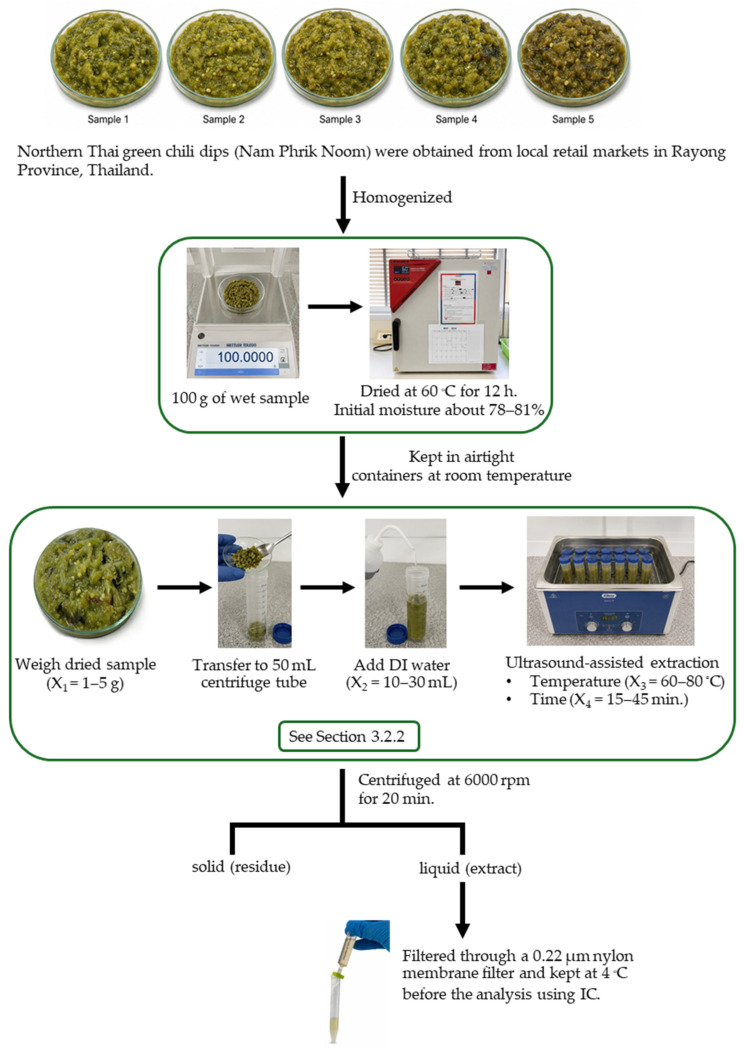
Schematic workflow of the developed UAE–IC method for benzoate determination in Northern Thai green chili dip.

An accurately weighed portion of the dried sample was transferred into a 50 mL polypropylene centrifuge tube and combined with the appropriate volume of deionized water to form a homogeneous suspension. UAE was performed in an ultrasonic bath (Elma Sonic P 30 H, Elma GmbH, Singen, Germany; 37 kHz, 380 W) at the temperatures and times as specified in the experimental design (see Section 3.2.2). After the sonication treatment, the extracts were allowed to cool to room temperature and then centrifuged at 6000 rpm for 20 min to separate the liquid (extract) and solid (residue) phases. The supernatant was decanted and filtered through a 0.22 µm nylon membrane filter (Whatman, Cytiva, UK) to remove the particulate matter. The filtrates were transferred into 1.5 mL auto sampler vials and kept at 4 °C before the analysis using IC. A schematic overview of the sample preparation and UAE procedure prior to ion chromatographic analysis is presented in Scheme 1.

#### 3.2.2. Optimization of UAE Conditions Using Response Surface Methodology

Statistical optimization of the UAE process was carried out using response surface methodology (RSM) with Box–Behnken design (BBD) under Minitab 18 software for Windows version 16.2.2. RSM is the best choice in solving multivariable problems as it allows a much smaller number of experiments to be conducted in comparison to full factorial designs, and is useful for simultaneously examining the effects and interactions of independent variables [30]. Moreover, BBD is more suitable than central composite and Doehlert designs for being rotatable or nearly rotatable, avoiding extreme factor-level combinations, and fitting the second-order polynomial model without the need of the experiments at the vertices of the design region [31]. Four variables that were identified as significant in the UAE process for the extraction of benzoate were sample mass (X_1_, 1–5 g), extraction solvent volume (X_2_, 10–30 mL), extraction temperature (X_3_, 60–80 °C) and extraction time (X_4_, 15–45 min). Three levels (−1, 0, +1) with equal intervals for each factor were considered and a BBD consisting of 27 experimental runs was developed, which also included three replicates at the center point to estimate pure error and check the reproducibility of the model (Table 5). The content of benzoate (mg kg^−1^, on a dry weight basis) was the response variable (Y) (denotes the benzoate concentration in the dried samples) and the second-order polynomial model (Equation (1)) was used to describe the relationship between independent variables and the response:

(1) $$Y=\beta_{0}+\sum_{i=1}^{3}\beta_{i}X_{i}+\sum_{i=1}^{3}\beta_{ii}X_{i}^{2}+\sum_{i=0}^{2}\sum_{j=i+2}^{3}\beta_{ij}X_{i}X_{j}$$

where Y is the predicted response; β_0_ is the intercept coefficient; β_i_, β_ii_, and β_ij_ represent the linear, quadratic, and interaction regression coefficients, respectively; and X_i_ and X_j_ denote the independent variables. All experimental runs were performed in randomized order to minimize systematic bias.

#### 3.2.3. Statistical Analysis and Model Evaluation

The ANOVA results were used to evaluate the significance of the regression model, as well as the effects of the independent variables and their interactions on benzoate extraction recovery. Statistical analyses were performed using Minitab for Windows (version 16.2.2) to generate regression equations, 2D contour plots, and to assess the relationships between the independent variables and the response. Three-dimensional response surface plots were generated using Python (version 3.12.13) to visualize the individual and interactive effects of the variables on benzoate recovery. The goodness-of-fit of the model was evaluated using the coefficient of determination (R^2^), adjusted R^2^, predicted R^2^, and the lack-of-fit test. The model was considered adequate when the signal-to-noise ratio (adequate precision) exceeded 4. Statistical significance of the regression model and its terms was established at *p* < 0.05. Finally, triplicate experiments were conducted under the optimum conditions predicted by the RSM model to validate its performance, and the experimental benzoate recovery values were compared with the predicted values to assess the validity and accuracy of the model.

#### 3.2.4. Ion Chromatographic Analysis

Benzoate was quantified by IC on a Metrohm 930 Compact IC Flex (Metrohm AG, Herisau, Switzerland) instrument fitted with a suppressed conductivity detector. Separation was performed on a Metrosep A Supp 5 anion-exchange column (250 mm × 4.0 mm i.d., 5 µm, Metrohm AG, Switzerland), which under carbonate-bicarbonate eluent conditions shows excellent selectivity for low-molecular-weight organic anions such as benzoate. An aqueous Na_2_CO_3_/NaHCO_3_ (both 3.20 mM) mobile phase was pumped isocratically at a flow rate of 1.00 mL min^−1^. The analytical column was maintained at 30 °C and the sample injection volume was 20 µL. All sample extracts and calibration standards were passed through 0.22 µm nylon filters prior to injection to prevent particulate-matter blockage of the column. Retention time matching with that of an authentic benzoate standard analyzed under identical IC conditions was used for peak assignment. External standard calibration (peak areas) was employed for quantitation.

#### 3.2.5. Method Validation

Linearity, sensitivity, precision, accuracy, LOD, and LOQ of the developed IC method were validated according to IUPAC recommendations. A series of standard solutions covering the working and calibration ranges was used for plotting the calibration curve. Linearity was studied by linear regression and calculation of the coefficient of determination (R^2^) as a measure of goodness of fit; a minimum value of 0.999 was deemed acceptable for quantitative analysis.

The limits of detection (LOD) and quantification (LOQ) were estimated based on signal-to-noise (S/N) ratios of 3 and 10, respectively, using analyte peak responses obtained under the optimized extraction conditions.

Method precision was evaluated through repeated analyses (n = 3 at each concentration level) of three independently prepared extracts from a representative chili dip sample, conducted both within a single day (intra-day precision) and over three consecutive days (inter-day precision). Precision was expressed as the relative standard deviation (%RSD), calculated as the ratio of the standard deviation (SD) to the corresponding mean value multiplied by 100. A %RSD value of ≤ 5% was considered indicative of acceptable method precision.

The accuracy of the method was investigated via the method of standard addition (or recovery). Aliquots of the working standard solution at a single spike level (20 mg kg^−1^) were spiked into a representative chili dip sample prior to extraction. After spiking, each sample was then subjected to the entire optimized procedure of UAE extraction and IC analysis. The absolute recoveries of the analyte were then calculated from the percentage ratio of the difference in concentrations with and without spiking to the spiked concentration. Acceptable recoveries ranging from 80 to 110% were used as the criteria for quantitative analysis.

#### 3.2.6. Greenness Assessment Using the AGREE Metric

The greenness assessment of the entire analytical workflow was performed using the freely accessible AGREE calculator, which generates a characteristic clock-like circular pictogram divided into 12 colour-coded segments.

## 4. Conclusions

In this study, an ultrasound-assisted extraction coupled with ion chromatography (UAE–IC) method was successfully developed and optimized for the determination of benzoate in Northern Thai green chili dip. The method demonstrated satisfactory analytical performance in terms of linearity, sensitivity, precision, and accuracy, meeting the acceptance criteria of IUPAC and AOAC. Application to commercial samples confirmed its suitability for routine analysis, and all samples contained benzoate levels below the maximum limits established by the Thai Food and Drug Administration and the Codex Alimentarius Commission.

The developed UAE–IC method provides a sensitive, reliable, and environmentally friendly approach for benzoate determination in condiment products. The method is potentially transferable to other high-matrix condiments following appropriate matrix-specific validation. However, multi-laboratory validation is recommended before routine regulatory implementation to confirm its reproducibility and robustness across different laboratories. Future studies may also explore its application to the simultaneous determination of multiple preservatives in food products.

## Figures and Tables

**Table 1:** Analysis of variance (ANOVA) and regression coefficients for the model predicting the extracted benzoate concentration.

Source	DF	SS	MS	*F* Value	*p* Value
Model	14	236,360	16,883	15.12	<0.001
*X*_1_	1	158,034	158,034	141.55	<0.001
*X*_2_	1	68	68	0.06	0.810
*X*_3_	1	7626	7626	6.83	0.023
*X*_4_	1	12,838	12,838	11.50	0.005
*X*_1_^2^	1	2303	2303	2.06	0.177
*X*_2_^2^	1	21,917	21,917	19.63	0.001
*X*_3_^2^	1	32,161	32,161	28.81	<0.001
*X*_4_^2^	1	5907	5907	5.29	0.040
*X*_1_ *X*_2_	1	4402	4402	2.31	0.102
*X*_1_ *X*_3_	1	2406	2406	2.15	0.168
*X*_1_ *X*_4_	1	522	522	0.47	0.507
*X*_2_ *X*_3_	1	6408	6408	5.74	0.034
*X*_2_ *X*_4_	1	1751	1751	1.57	0.234
*X*_3_ *X*_4_	1	0	0	0.00	0.994
Residual error	12	13,398	1116		
Lack-of-Fit	10	11,253	1125	1.05	0.582
Pure error	2	2145	1072		
Total	26	249,757			
		R^2^ = 0.9464		Adjust R^2^ = 0.8838	

DF—degree of freedom; SS—sum of square; MS—mean square; *F*—*F*-statistics test to determine significance; *p*—probability value.

**Table 2:** Analytical performance of the developed UAE–IC method for benzoate determination.

Parameter	Result
Linear range (mg L^−1^)	0.50–100
Equation	y = 1.251x + 0.076
Correlation coefficient (R^2^)	0.9993
LOD (mg L^−1^)	0.305
LOQ (mg L^−1^)	1.070
Precision (%RSD, n = 11)	0.31–6.37
Recovery (%)	85.50–107.80

**Table 3:** Determination of benzoate in chili dip samples using the UAE-IC method and recovery study.

Sample	Spike (mg kg^−1^)	Benzoate Concentration ^a^		Recovery (%)
		(mg kg^−1^ Dry Weight, C_dw_)	(mg kg^−1^ Wet Weight, C_ww_) ^b^	
Chili dip 1	0	321.86 ± 2.88	65.67 ± 1.87	85.50 ± 2.16
	20	338.96 ± 1.95	-	
Chili dip 2	0	501.07 ± 2.28	103.23 ± 3.59	107.80 ± 2.25
	20	523.20 ± 3.16	-	
Chili dip 3	0	300.73 ± 2.38	62.25 ± 2.89	87.50 ± 3.01
	20	318.23 ± 2.54	-	
Chili dip 4	0	263.00 ± 1.55	54.33 ± 1.68	105.60 ± 1.96
	20	284.12 ± 3.82	-	
Chili dip 5	0	464.77 ± 1.23	96.85 ± 4.26	89.05 ± 2.83
	20	482.58 ± 2.07	-	

^a^ Values are presented as means ± SD (n = 3). ^b^ Calculated from C_ww_ = C_dw_ × [1 − (Moisture content/100)].

**Table 4:** Comparison of the proposed UAE–IC method with other available methods for benzoate determination in foods.

Method/Extraction	Solvent Consumption	Matrix	LOD (mg kg^−1^)	Recovery (%)	Precision (%RSD)	Reference
IC-CD/UAE	Solvent-free (water)	Northern Thai green chili dip	0.305	85.50–107.80	0.31–6.37	This work
SPE-IC-CD/SPE	Aqueous	milk	-	≈90–105	<5	[20]
CIC-CD/LLE/SLE	Alkaline aqueous	foods and beverages	1.6–4.1	90.2–105.8	0.3–3.7	[21]
RP-HPLC-DAD/UAE	Ethanol/water	multiple food matrices	-	84.0–100.8	-	[29]
GC-MS/Headspace	Solvent-lean	tomato paste, ketchup and sauces	1.50	98.5–100.3	2.8–4.30	[30]
GC-FID/HS-SPME	Solvent-free	soy and fish sauces, pickled vegetable, shrimp paste	1.10	83.0–109.0	4.5–6.0	[31]
CE-UV/LLE	Alkaline aqueous	sauces	0.22	81.0–105.0		[32]

SLE: solid–liquid extraction; LLE: liquid–liquid extraction; SPE: solid phase extraction; HS-SPME: head space solid phase microextraction; CZE-UV: capillary zone electrophoresis with ultraviolet detection; RP-HPLC-DAD: reversed phase-high performance liquid chromatography with diode array detection; GC-MS: gas chromatography with mass spectrometric detection; GC-FID: gas chromatography with flame ionization detection; IC-CD: ion chromatography with conductivity detection; CE-UV: capillary electrophoresis with ultraviolet detection; CIC-CD: capillary ion chromatography with conductivity detection.

**Table 5:** Box–Behnken experimental design matrix for four UAE process variables (X_1_–X_4_) across 27 runs, with measured and model-predicted benzoate contents.

Run	Factors				Benzoate Concentration (mg kg^−1^ Dry Weight, C_dw_)	
	Sample Mass (X_1_, g)	Solvent Volume (X_2_, mL)	Temperature (X_3_, °C)	Extraction Time (X_4_, min)	Measured Value	Predicted Value
1	1	20	70	45	135.10	149.57
2	3	20	80	15	318.90	286.78
3	3	30	70	15	234.10	256.44
4	5	20	70	15	441.10	444.50
5	3	20	70	30	311.50	339.67
6	5	30	70	30	403.50	405.09
7	5	20	80	30	407.90	405.73
8	3	10	60	30	119.40	130.30
9	3	20	60	15	214.90	236.11
10	5	20	60	30	316.80	306.26
11	3	20	70	30	375.60	339.67
12	3	20	60	45	154.90	170.94
13	3	10	70	15	318.50	293.54
14	1	20	80	30	118.40	127.16
15	3	30	70	45	209.70	232.88
16	5	10	70	30	334.00	333.99
17	3	30	80	30	178.50	185.47
18	3	10	80	30	204.90	260.77
19	3	20	80	45	258.40	221.11
20	5	20	70	45	348.50	356.23
21	3	30	60	30	253.10	215.10
22	1	30	70	30	125.30	109.23
23	1	10	70	30	188.50	170.83
24	1	20	70	15	182.00	192.13
25	1	20	60	30	125.40	125.79
26	3	20	70	30	331.90	339.67
27	3	10	70	45	210.40	186.28

## Data Availability

The original contributions presented in this study are included in the article/Supplementary Material. Further inquiries can be directed to the corresponding author.

## Supplementary Materials

The following supporting information can be downloaded at: https://www.mdpi.com/article/10.3390/molecules31132259/s1.

## References

[1] World Health Organization (2019). Evaluation of Certain Food Additives: Eighty-Sixth Report of the Joint FAO/WHO Expert Committee on Food Additives 2019.

[2] Davidson P.M., Sofos J.N., Branen A.L. (2005). Antimicrobials in Food 2005.

[3] EFSA ANS Panel (EFSA Panel on Food Additives and Nutrient Sources Added to Food) (2016). Scientific Opinion on the re-evaluation of benzoic acid (E 210), sodium benzoate (E 211), potassium benzoate (E 212) and calcium benzoate (E 213) as food additives. EFSA J..

[4] Mehta N., S J., Kumar P., Verma A.K., Umaraw P., Khatkar S.K., Khatkar A.B., Pathak D., Kaka U., Sazili A.Q. (2022). Ultra-sound-Assisted Extraction and the Encapsulation of Bioactive Components for Food Applications. Foods.

[5] Lučić M., Onjia A. (2025). Ultrasound-Assisted Microextraction for Food Chemical Contaminant Analysis: A Review. Processes.

[6] Lou Z., Cheng X., Dong J., Dai W., Chen X., Wang H. (2026). The antibacterial mechanism and cell damage mechanism of natural antibacterial agents from different sources and their applications in food. J. Food Meas. Charact..

[7] Issa H.M., Mohammed D.H. (2025). A Critical Review on the Journey of Benzoic Acid in the Pharmaceutical Industry from Manufac-turing Processes Through Various Uses to Disposal: An Environmental Perspective. Environ. Anal. Health Toxicol..

[8] Ministry of Public Health (2020). Notification of the Ministry of Public Health (No. 418) B.E. 2563 (2020) on the Standards for Food Additives in Food.

[9] Hejazi L., Mahboubi-Rabbani M., Mahdavi V., Alemi M., Khanniri E., Bayanati M. (2024). A critical review on sodium benzoate from health effects to analytical methods. Results Chem..

[10] Walczak-Nowicka Ł.J., Herbet M. (2022). Sodium benzoate—Harmfulness and potential use in therapies for disorders related to the nervous system: A review. Nutrients.

[11] Pongsetkul J., Benjakul S. (2021). The use of sodium benzoate on shelf-life and quality attributes of dried chili fish paste stored in different packaging containers. Foods.

[12] Khunsee A., Kaewsritong J., Srikaeo K. (2024). Clean label approach for condiment sauces: A case in Thai style sauces. Multidiscip. Sci. J..

[13] Lama-Muñoz A., Contreras M.D.M. (2022). Extraction systems and analytical techniques for food phenolic compounds: A review. Foods.

[14] Mota F.J., Ferreira I.M., Cunha S.C., Oliveira M.B.P. (2003). Optimisation of extraction procedures for analysis of benzoic and sorbic acids in foodstuffs. Food Chem..

[15] Chemat F., Rombaut N., Sicaire A.-G., Meullemiestre A., Fabiano-Tixier A.-S., Abert-Vian M. (2017). Ultrasound assisted extraction of food and natural products. Mechanisms, techniques, combinations, protocols and applications. A review. Ultrason. Sono-chem..

[16] Lv J.-M., Gouda M., Zhu Y.-Y., Ye X.-Q., Chen J.-C. (2021). Ultrasound-Assisted Extraction Optimization of Proanthocyanidins from Kiwi (*Actinidia chinensis*) Leaves and Evaluation of Its Antioxidant Activity. Antioxidants.

[17] Madondo N.I., Chetty M. (2022). Anaerobic co-digestion of sewage sludge and bio-based glycerol: Optimisation of process variables using one-factor-at-a-time (OFAT) and Box-Behnken Design (BBD) techniques. S. Afr. J. Chem. Eng..

[18] Wang X., Wu Y., Chen G., Yue W., Liang Q., Wu Q. (2013). Optimization of ultrasound assisted extraction of phenolic compounds from Sparganii rhizoma with response surface methodology. Ultrason. Sonochem..

[19] Anaya-Esparza L.M., Aurora-Vigo E.F., Villagrán Z., Rodríguez-Lafitte E., Ruvalcaba-Gómez J.M., Solano-Cornejo M.Á., Zamora-Gasga V.M., Montalvo-González E., Gómez-Rodríguez H., Aceves-Aldrete C.E. (2023). Design of experiments for optimizing ultrasound-assisted extraction of bioactive compounds from plant-based sources. Molecules.

[20] Wang Z.-H., Xia J.-F., Zhao F.-Y., Han Q., Guo X.-M., Wang H., Ding M.-Y. (2013). Determination of benzoic acid in milk by solid-phase extraction and ion chromatography with conductivity detection. Chin. Chem. Lett..

[21] D’Amore T., Di Taranto A., Berardi G., Vita V., Iammarino M. (2021). Going green in food analysis: A rapid and accurate method for the determination of sorbic acid and benzoic acid in foods by capillary ion chromatography with conductivity detection. LWT.

[22] Pena-Pereira F., Wojnowski W., Tobiszewski M. (2020). AGREE—Analytical GREEnness metric approach and software. Anal. Chem..

[23] Deng Y., Wang W., Zhao S., Yang X., Xu W., Guo M., Xu E., Ding T., Ye X., Liu D. (2022). Ultrasound-assisted extraction of lipids as food components: Mechanism, solvent, feedstock, quality evaluation and coupled technologies—A review. Trends Food Sci. Technol..

[24] Stańczyk M., Boruń A., Jóźwiak M. (2019). Conductance studies of aqueous solutions of sodium salts of selected benzoic acid deriv-atives at temperatures from (288.15 to 318.15) K. J. Mol. Liq..

[25] Chemat F., Vian M.A., Cravotto G. (2012). Green extraction of natural products: Concept and principles. Int. J. Mol. Sci..

[26] Briliantama A., Oktaviani N.M.D., Rahmawati S., Setyaningsih W., Palma M. (2022). Optimization of ultrasound-assisted extraction (UAE) for simultaneous determination of individual phenolic compounds in 15 dried edible flowers. Horticulturae.

[27] Gören A.C., Bilsel G., Şimşek A., Bilsel M., Akçadağ F., Topal K., Ozgen H. (2015). HPLC and LC–MS/MS methods for determina-tion of sodium benzoate and potassium sorbate in food and beverages: Performances of local accredited laboratories via profi-ciency tests in Turkey. Food Chem..

[28] Latimer G.W., Horwitz W. (2019). Official Methods of Analysis.

[29] Chakraborty A., Jayaseelan K. (2025). Environmentally Sustainable Reverse Phase-High Performance Liquid Chromatography Method for the Simultaneous Estimation of Sodium Benzoate and Potassium Sorbate in Food Matrices Utilizing Green Ultrasound Assisted Extraction: An Analytical Quality by Design Approach with Greenness, Blueness, and Whiteness Assessment. Microchem. J..

[30] Destanoğlu O. (2024). Investigation of Benzoic Acid and Sorbic Acid Concentrations in Tomato Paste, Pepper Paste, Ketchup, Mayonnaise, and Barbeque Sauce Samples by Headspace Gas Chromatography–Mass Spectrometry. Turk. J. Chem..

[31] Tungkijanansin N., Alahmad W., Nhujak T., Varanusupakul P. (2020). Simultaneous Determination of Benzoic Acid, Sorbic Acid, and Propionic Acid in Fermented Food by Headspace Solid-Phase Microextraction Followed by GC-FID. Food Chem..

[32] Aung H.-P., Pyell U. (2016). In-Capillary Derivatization with o-Phthalaldehyde in the Presence of 3-Mercaptopropionic Acid for the Simultaneous Determination of Monosodium Glutamate, Benzoic Acid, and Sorbic Acid in Food Samples via Capillary Electrophoresis with Ultraviolet Detection. J. Chromatogr. A.

[33] Timofeeva I., Kanashina D., Kirsanov D., Bulatov A. (2018). A heating-assisted liquid-liquid microextraction approach using menthol: Separation of benzoic acid in juice samples followed by HPLC-UV determination. J. Mol. Liq..

[34] Quigley A., Cummins W., Connolly D. (2016). Dispersive liquid-liquid microextraction in the analysis of milk and dairy products: A review. J. Chem..

[35] Wojnowski W., Tobiszewski M., Pena-Pereira F., Psillakis E. (2022). AGREEprep–analytical greenness metric for sample preparation. TrAC Trends Anal. Chem..

