# Supplementary material for: Ultrasound-Assisted Extraction Coupled with Ion Chromatography for Benzoate Determination in Northern Thai Green Chili Dip: Box–Behnken Optimization, Greenness Assessment, and Application to Commercial Samples
Source: Molecules. 2026 Jun 26;31(13):2259. doi: 10.3390/molecules31132259 (PMC13362715; doi:10.3390/molecules31132259)
Supplement: Supplementary file 1 [file molecules-31-02259-s001.zip › molecules-4276295-supplementary.pdf]

Supporting Information

Ultrasound-Assisted Extraction Coupled with Ion Chromatography for Benzoate Determination in Northern Thai Green Chili Dip: Box–Behnken Optimization, Greenness Evaluation, and Application to Commercial Samples

Manuscript ID: molecules-4276295, Molecules (MDPI)

S1. Detailed AGREE greenness assessment

The AGREE software reports an overall greenness score together with a colour-coded clock pictogram rather than numerical values for each individual principle. Accordingly, Table S1 documents the input selection made for each of the twelve principles of green analytical chemistry, for both the proposed UAE–IC method and the conventional LLE-HPLC-UV method. The colour shown in parentheses for each selection correspond to the colour of the corresponding segment in the AGREE clock pictograms (Figure 3 of the main text); the overall scores were 0.45 (UAE–IC) and 0.27 (conventional LLE-HPLC-UV), a 67% relative improvement.

Table S1. AGREE input selections for each of the twelve green-analytical-chemistry criteria for the proposed UAE–IC method (overall score 0.45) and the conventional LLE–HPLC–UV method (overall score 0.27). The colour in parentheses indicates the colour of the corresponding segment in the AGREE pictogram (Figure 3).

Pr.	GAC criterion	Proposed UAE–IC method (overall 0.45)	Conventional LLE–HPLC–UV (overall 0.27)
1	**Sample treatment**	off-line external pre-treatment; batch analysis with a reduced number of steps **(orange)**	off-line external pre-treatment; batch analysis with a large number of steps **(red)**
2	**Sample size**	input amount 5.0 g **(gold)**	input amount 0.5 g **(lightgreen)**
3	**Device positioning**	off-line **(red)**	off-line **(red)**
4	**Number of steps**	4 distinct steps **(lightgreen)**	3 or fewer distinct steps **(lightgreen)**
5	**Automation & miniaturization**	manual; not miniaturized **(red)**	manual; not miniaturized **(red)**
6	**Derivatization**	none selected **(green)**	none selected (with matrix parameter variance) **(gold)**
7	**Analytical waste**	45 mL per run **(orange)**	50 mL per run (toxic organic-solvent waste) **(red)**
8	**Analytes & throughput**	1 analyte; 3 samples per hour **(orange)**	1 analyte; 10 samples per hour **(gold)**
9	**Energy consumption**	LC/ultrasound-assisted extraction **(gold)**	LC + hot-plate solvent evaporation (>150 min) **red)**

10	**Bio-based reagents**	none of the reagents from bio-based sources **(red)**	none of the reagents from bio-based sources **(red)**
11	**Toxic reagents**	no toxic reagents/solvents (deionized water only) **(green)**	toxic solvents involved: methanol, acetonitrile, chloroform **(orange)**
12	**Operator safety**	no threats selected **(green)**	threats: toxic to aquatic life, highly flammable, corrosive **(orange)**
	**Overall AGREE score**	**0.45**	**0.27**

The principal greenness advantage of the proposed method arises from the elimination of toxic organic solvents (Principle 11) and the associated operator-safety benefit (Principle 12), together with the lower-hazard aqueous waste stream (Principle 7) and the reduced energy demand without a solvent-evaporation step (Principle 9). The conventional method scores more favorably on sample amount (Principle 2) and sample throughput (Principle 8), while both methods are similarly constrained by off-line analysis (Principle 3), manual and non-miniaturized sample preparation (Principle 5), and the absence of bio-based reagents (Principle 10).
